# Résultats fonctionnels des lésions des tendons fléchisseurs de la main: à propos de 90 cas

**DOI:** 10.11604/pamj.2013.15.1.2228

**Published:** 2013-05-01

**Authors:** Hassan Boussakri, Mohamad Azarkane, Mohamad Elidrissi, Mohamad Shimi, Abdelhalim Elibrahimi, Abdelmajid Elmrini

**Affiliations:** 1 Service de chirurgie osteoarticulaire B4 Fès. CHU Hassan II, 30000 Fès, Maroc

**Keywords:** Tendons fléchisseurs, main, réparation, résultats fonctionnels, flexor tendons, hand, repair, functional results

## Abstract

Les auteurs rapportent une série de 90 patients, présentant une section tendineuse des fléchisseurs de la main, et suivis avec un recul moyen de 8 mois (min: 2 mois; max: 13 mois). La lésion était localisée dans 12% des cas en zone I, 46% des cas en zone II, 2% en zone III et 25% des cas en zone IV et V. Pour le pouce (13 patients), 10 cas en zone T2 et 3 cas en zone T3. La technique opératoire utilisée était les sutures tendineuses en cadre de Kessler modifié, associée à un surjet épitendineux. Nous avons obtenus 54% de très bons résultats, 34% de résultats moyens et 12% de mauvais résultats. Pour le pouce les résultats semble moins bons avec un taux de résultats médiocre de 48%. Certes les chiffres de cette série sont moins bons que ceux des autres séries publiées dans la littérature. Les facteurs influençant les résultats sont d'abord l'utilisation d'immobilisation postopératoire systématique ainsi que le mécanisme d'agression, et la localisation à la zone II et au pouce. Les complications mécaniques sont représentées par 7% de rupture, toutes au niveau de pouce, et 31% des adhérences tendineuses (soit 30 cas), dont 19 en zone II, l'infections (22%) et 4% des cas d'algodystrophies.

## Introduction

Les lésions des tendons fléchisseurs ont une gravité fonctionnelle souvent sous-estimée par les patients, voire les médecins eux-mêmes. Leur prise en charge commence dès l'arrivée du patient dans la structure médicale ou chirurgicale d'accueil [1]. La réparation de ces lésions constitue un véritable enjeu fonctionnel. Il a été démontré que c'est au prix d′une chirurgie minutieuse suivie d′une rééducation et d′un appareillage postopératoire bien réalisé que l′on peut procurer des résultats fonctionnels de bonne qualité [2]. Le pronostic des lésions des tendons fléchisseurs est essentiellement lié aux cinq zones topographiques et aux autres lésions associées [3]. La durée de l′arrêt du travail est un critère de résultat important en termes de coût pour la société et de réinsertion professionnelle pour les patients [4]. Le but de notre travail était, à partir d′une étude rétrospective, d’évaluer les résultats fonctionnels de réparation primaire des tendons fléchisseurs de la main ainsi que la répartition topographique de ces lésions et l′impact socioéconomique de cette pathologie.

## Méthodes

Il s′agit d′une étude rétrospective, couvrant la période de Janvier 2010 à Mai 2012, concernant tous les patients traités d′au moins une lésion des fléchisseurs de la main. Ces patients ont été colligés au service de chirurgie ostéo-articulaire B4 du CHU de FES.

Notre série intègre 90 patients dont 83 hommes et 7 femmes. Le recul recul moyen était de 8 mois (2 - 13 mois). Les données ont été collectée pour chaque malade à l'aide d'une grille préétablie comportant les variables suivantes: sexe, âge, nature dominante de la main lésée, type d′accident, topographie lésionnelle (en utilisant la classification de la Fédération Internationale qui repartit le niveau lésionnel en 5 zones pour les doigts longs et 3 zones pour le pouce), nature de traumatisme, nombre des doigts touchés, numéro du doigt, lésions associées, profession et protocole thérapeutique.

Plusieurs méthodes d′évaluation permettent d'apprécier les résultats des réparations de tendons fléchisseurs parmi lesquelles la classification de la Société Américaine de Chirurgie de la Main (T. A. M), la méthode de Strickland et la classification de Buck-Gramcko. Chacune de ces méthodes présente des avantages mais aussi des points faible [5, 6].

Dans notre série, l’évaluation était basée sur la cotation de la Fédération Internationale des Sociétés de Chirurgie de la Main pour les doigts longs, et la cotation de Tubiana pour le pouce [7].

Nous n′avons retenu que les lésions des fléchisseurs de la main. Ont été exclus de cette série tous les cas comportant des lésions tendineuses opérées chez les enfants de moins de 15 ans, et les lésions plus complexes osseuses et nerveuses pouvant constituer en elles-mêmes un facteur limitant la mobilité finale-surtout au niveau du poignet: zone IV et V, ainsi que les dossiers médicale inexploitable.

### Technique (Figure 1, Figure 2, Figure 3)

La voie d′abord utilisée a été la plaie elle-même agrandie à ses extrémités par des incisions obliques en L (en zigzag), en respectant les règles de la chirurgie de la main [8]. L'ouverture du canal digital doit être conduite de façon atraumatique et orienté en fonction du type de la plaie, épargnant les poulies (en particulier A2 et A4). On explore systématiquement les pédicules vasculo-nerveux et on accorder autant de temps et d′attention. Après repérage de tendon et immobilisation des 2 extrémités par des aiguilles intradermiques, lorsque la récupération du bout proximal n'est pas possible on utilise la mise en flexion du doigt ou du poignet ainsi que le massage d'amont en aval des masses musculaires qui peut suffire dans certains cas à extérioriser le tendon. Dans le cas contraire, il faut utiliser une technique de cathétérisme du canal digital.

**Figure 1:**
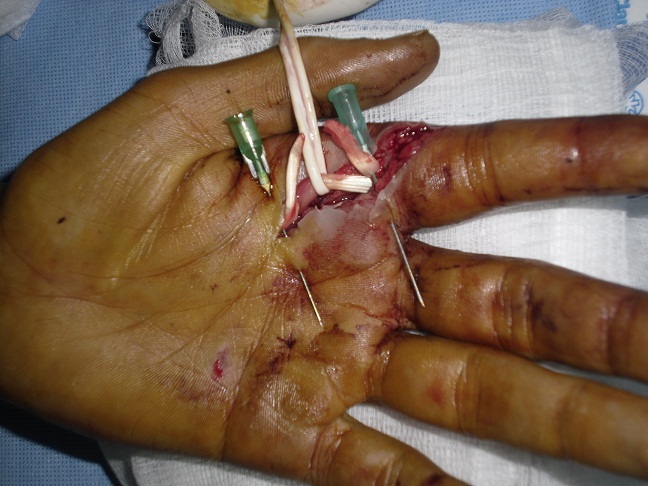
La technique de réparation après section des tendons fléchisseurs: un point en cadre de type Kessler. Vue 1

**Figure 2:**
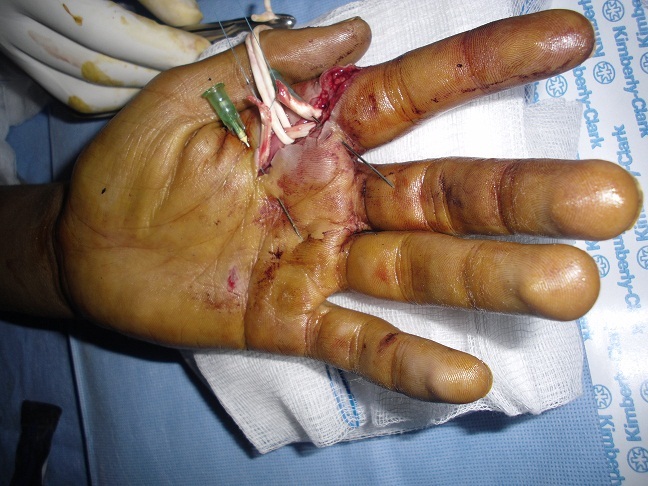
La technique de réparation après section des tendons fléchisseurs: un point en cadre de type Kessler. Vue 2

**Figure 3:**
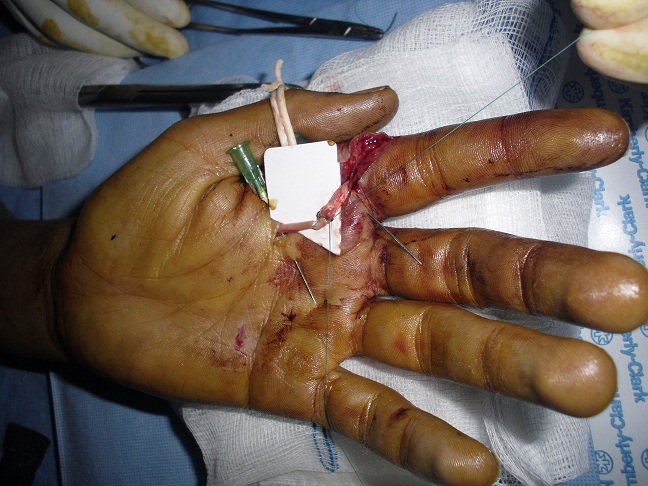
La technique de réparation après section des tendons fléchisseurs: un point en cadre de type Kessler. Vue 3

Nous avons utilisée des sutures tendineuses en cadre de Kessler modifié au fil fin non résorbable de Nylon 4:0, avec le nœud enfoui dans la tranche de section, l'affrontement est complété par un surjet epitendineux. Nous avons utilisé cette technique chez tous les malades sauf pour les lésions de zone 1 et T1 de pouce pour lesquels on a utilisé la fixation en rappel. La régularisation de la zone de suture est primordiale afin d’éviter l′accrochage du tendon lors du passage sous les poulies.

Suivant le type de traumatisme d′une part et le profil psychologique du malade d′autre part, on décide une immobilisation pour une protection des sutures par la mise en place d′une attelle postérieure à 45° de flexion du poignet, 80° de flexion des métacarpo-phalangiennes et 15° de flexion des inter-phalangiennes. Alors que pour le pouce, la protection est assurée par une attelle antibrachio-palmaire, poignet fléchi à 30° associée à une attelle dorsale protégeant la colonne du pouce [9]. L'immobilisation est maintenue un minimum de 4 semaines, le patient étant revu 2 fois, à j + 7 pour détecter une complication précoce et apprécier sa compréhension du programme de rééducation et sa coopération, et à 4 semaines pour commencer la rééducation.

## Résultats

Entre Janvier 2010 et Mai 2012, 90 blesses ont été traitées, 92% des blesses étaient des hommes (83 hommes et 7 femmes). L’âge moyen était de 25 ans, avec des extrêmes allant de 16 à 42 ans. II y avait une nette prédominance de lésions à la main gauche (58 gauches contre 32 droites). La main dominante n′était pas plus souvent blessée que l′autre (49%). Tous les patients ont été opérés dans les 24 premières heures après le traumatisme. Le délai moyen entre le traumatise et l'admission aux urgences était de 3 heures. Concernant le mécanisme de lésion, dans 72% il s′agissait d′agression par arme blanche (64 patients), 20% accidents domestiques (éclat de verre dans 09 patients et 10 coupures domestiques par couteau), et 8% accident de travail (7 patients). L'activité professionnelle était repartie de la manière suivante: sans-emplois 85%, travailleurs manuels 13% et étudiants 2%.

Selon la classification topographique de la Fédération Internationale des Sociétés de Chirurgie de la Main (IFSSH), il y avait 11 lésions en zone I (soit 12%), 42 en zone II (soit 46%), 2 en zone III (soit2%) et 22 en zone IV et V (soit 25%) pour les doigts longs. Pour le pouce: 13 patients dont 10 cas en zone T2 et 3 cas en zone T3. Concernant les lésions associées on a noté 12 cas d'atteinte un nerf, 7 cas d'atteinte artérielle et 10 cas d'atteinte articulaire.

### Au niveau des doigts longs

Quant à la répartition des blessures entre les 5 doigts de la main, elles sont homogènes, puisque ne variant qu'entre 20% pour le 2eme doigt, 23% pour le 3eme doigt, 38% au 4eme doigt et 19% pour le 5eme doigt. L′atteinte de plusieurs doigts à la fois était relevée dans 9 cas. 42 sections tendineuses en zone II pour les doigts longs chez 53 blessés ont été réparées avec des 11 lésions de type I; le pédicule vasculo-nerveux était lésé dans 40% des cas. Dans 42 cas, les deux tendons fléchisseurs sont sectionnés et dans 13 cas seul le fléchisseur profond est sectionné, toujours en zone II. Les résultats avec un recul moyen de 8 mois, montrent 54% de bons résultats, 38% de moyens et 8% de mauvais résultats. En ce qui concerne les cas de mauvais résultats après suture primitive, vue le profil psychologique des malades, une re-intervention n'a pas été préconisé.

### Au niveau du pouce

Sur les 13 sections; 10 cas en zone T2 et et 3 cas en zone T3. Les résultats montrent 52% bons et très bons résultats, 48% de résultat médiocre (6 CAS).

### Lésions tendineuses en dehors des doigts long et du pouce Zone 3/Zone 4 /Zone 5

**Figure 4:**
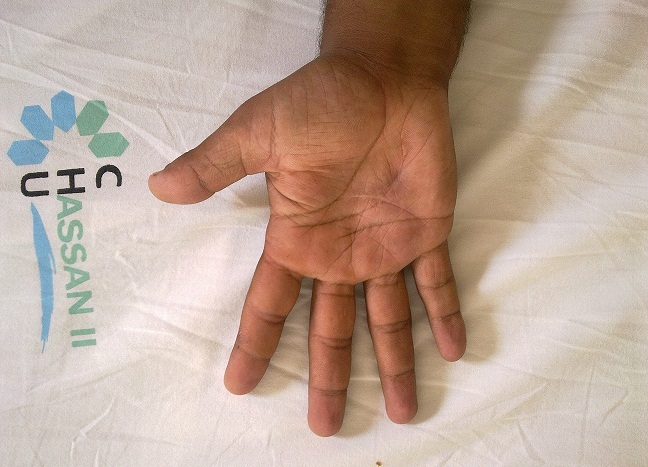
Résultats satisfaisants d'une lésion des fléchisseurs au niveau de la zone III. Vue 1

**Figure 5:**
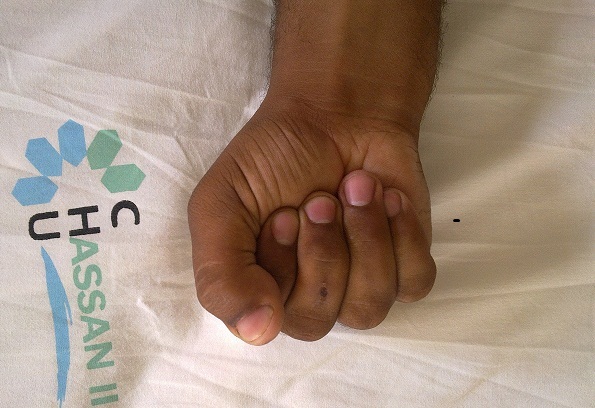
Résultats satisfaisants d'une lésion des fléchisseurs au niveau de la zone III. Vue 2

**Figure 6:**
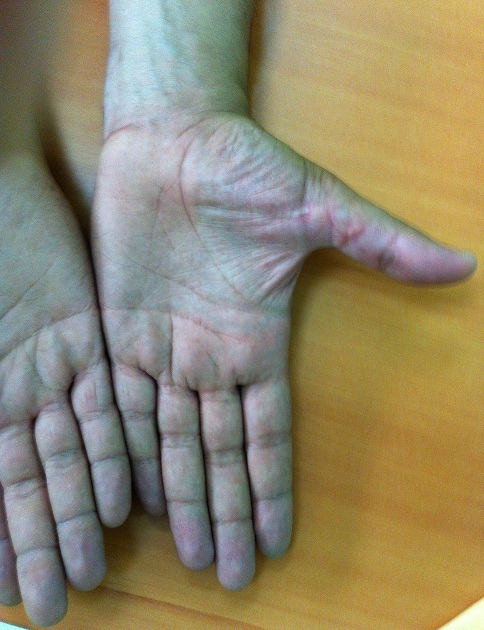
Rupture secondaire de lésion de long fléchisseur de pouce (zone T3). Vue 1

**Figure 7:**
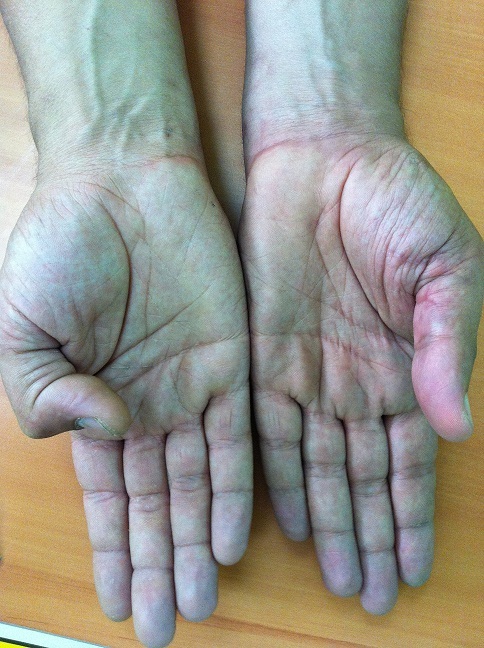
Rupture secondaire de lésion de long fléchisseur de pouce (zone T3). Vue 2

Globalement, nous avons obtenu 54% de très bons résultats, 28% de résultats moyens et 18% de mauvais résultats. Si on intègre les adhérences dans les complications, le taux est de 31% soit 30 adhérences (dont 19 en zone II), infections dans 22% (21 cas) juguler par antibiothérapie, 6 ruptures toutes au niveau de pouce et 4 algodystrophies (4%). Les conséquences de ces plaies ont été sévères, puisque les patients ont interrompu leur activité professionnelle pendant 3 à 7 mois. Les résultats de ce notre série sont résumés dans le Tableau 1, et Tableau 2 ainsi que les Figure 4, Figure 5, Figure 6, Figure 7

**Tableau 1:** Résultat clinique de la série

Paramètre	Données
Série	90 patients
Âge	moyenne25 ans (16–42 ans)
Sexe	H : 92% (soit 83 patients) F : 8% (soit 07 patients)
Main dominante	49% des cas
Main lésée	Droite : 42%( 32 patients) Gauche : 64% (58 patients)
Plusieurs doigts atteints	19 cas
Lésions associées	Atteinte un nerf : 12 cas Atteinte une artère : 7 cas Atteinte une articulation: 10 cas
agression : arme blanche	72% (64 patients)
Accidents domestiques	20% (19 patients)
Accident du travail	8% (7 patients)
Chômeurs	85% (76 patients)
Travailleur manuel: agriculteur/commerçants	13% (12 patients)
Etudiants	2% (2 patients)
Délai entre traumatise et admission à l'urgence:	3 heures
Délai entre admission aux urgences et admission au bloc opératoire	24 heures

**Tableau 2:** Répartition topographique des lésions tendineuse de la main

Topographie	Nombre de patients (%)
Zone 1	11 (12%)
Zone 2	42 (46 %)
Zone 3	2 (2%)
Zone 4	17 (19%)
Zone 5	5 (6%)
Pouce	13 (14%)

## Discussion

La main traumatique constitue un motif de consultation fréquent au service d'urgence. Entre le janvier 2010 et le Mai 2012, 475 plaies de la main ont été opérées au bloc opératoire des urgences du CHU Hassan 2 de Fès (service des statistique chu Hassan 2 Fès).

La réparation primaire des plaies des tendons est une chirurgie récente. En effet Bunnel [10], recommandait le recours à la greffe tendineuse en raison des adhérences que provoquait cette réparation. C'est Verdan [11] qui a préconisé la réparation primaire des tendons tout en acceptant des zones anatomiques ou les résultats pouvaient être plus aléatoires. Cette classification est adoptée aujourd′hui par la Fédération Internationale avec quelques aménagements.

La majorité de nos patients avec lésions des fléchisseurs de la main est jeune avec un âge moyen est de 25 ans. Les agressions sont les premiers pourvoyeurs, contrairement aux données de la littérature ou en trouve les accidents de travail et les accidents de loisir ou domestiques [3–12].

Notre série ne diffère pas de celles de la littérature pour ce qui concerne les autres variables comme l′âge, la répartition selon le sexe (prédominance masculine), le coté atteint, le coté prédominant [3–12].

Les procèdes de suture sont variés et sont presque équivalents du point de vue résistance à la traction, les fils prennent appui sur les cloisons conjonctives fibreuses du tendon. Ils doivent préserver le glissement du tendon par un affrontement parfait des extrémités tendineuses en utilisant le matériel de suture le plus fin possible. Le tendon est un organe vivant et l′intervention chirurgicale ne doit pas ajouter de lésion au traumatisme initial [9].

La prise en charge post-opératoire de ces lésions est aussi importante que la technique chirurgicale. Tous les auteurs s'accord sur l'intérêt de la mobilisation post-opératoire précoce (Kleinert-Duran, combinaison des deux méthodes proposé par Chow) [13–15]. Il est vrai que celle-ci n'a pas réservée aux patients peu coopérants, mais aussi aux lésions comportant une composante d′écrasement [14]. C'est le cas dans notre série vue que la majorité de nos patients ne sont pas capables de comprendre les principes de la mobilité contrôlée, c'est pour cela que nous recommandons l′immobilisation postopératoire des articulations.

Il est admis que la comparaison en matière de chirurgie des tendons fléchisseurs est extrêmement difficile, à cause de l′hétérogénéité des séries et des différentes méthodes d′évaluation des résultats. Les types de lésions sont rarement identiques, par conséquent le geste chirurgical ne peut pas être toujours reproductible, la littérature est riche en cotations pour l′évaluation des résultats, et dans un but d′unification, la Société Américaine de Chirurgie de la Main a obtenu un consensus; Jensen [16] après comparaison des différentes méthodes de mesure (Strickland, Kleinert, Strickland modifié, Buck-Gramcko), a donné la préférence au TAM; c′est cette cotation que nous avons adoptée sauf pour le pouce.

La multiplicité des classifications utilisées rend bien entendu difficile la comparaison des séries publiées. Les chiffres de bons résultats oscillent entre 60 et 70% avec un pourcentage supérieur quand on ne conserve que les plaies franches [19]. Globalement, les résultats selon les séries varient entre 70% et 90% d'excellents résultats, le taux de rupture moyen oscille entre 4 et 10%. Pour le pouce les résultats semblent moins bons, avec une fourchette de 53 à 80 % d'excellents et bons résultats et un taux de rupture moyen entre 3 et 17% [17, 18], Alors que dans les travaux Merle M et al, les auteurs ont trouver 72,2 % de bons ou très bons résultats sur 23 lésions en zone II ou III [19].

Certes, les chiffres de cette série sont moins bons que ceux de littérature; nous avons essayé de déterminer les facteurs susceptibles d′avoir une incidence sur le résultat final de notre étude et nous avons retenu d′abord l'utilisation systématique d'immobilisation postopératoire. Dans une étude publiée par V Berard et al, les auteurs ont trouvé que la mobilisation semi-active selon Kleinert a donné 65% de bons résultats contre 54% en cas d′immobilisation post-opératoire [20]; aussi le statut économique précaire et l'absence de couverture sociale, puisque 85 % de nos patients sont sans-emplois. Par ailleurs l'aspect psychologique de nos patients jouait un rôle non négligeable dans le résultat final notamment pour l'absentéisme et la décision d'immobilisation postopératoire ainsi que le suivie de protocole de rééducation.

Par ailleurs la zone des lésions est un facteur qui influence les résultats fonctionnels; la zone I, II et le pouce sont les zones les plus difficile, et la prise en charge post-opératoire a le plus a jouer que d'autres localisations. Les autres facteurs qui semblent avoir un impact sur les résultats sont les lésions associées: notamment l′atteinte nerveuse et vasculaire.

Par ailleurs d'autres facteurs n′étaient pas significativement déterminants. Il s′agit du sexe, de l’âge du patient et de la technique de suture (puisque tous les malades ont été opéré par la même technique de suture) et le délai opératoire qui a été de moins de 24 heures. Dans le travail de V Berard [20], Les patients ayant un délai opératoire supérieure à 48 heures obtiennent 76 % de bons résultats contre seulement 55% pour les réparations en urgence absolue. Les auteurs ont expliqué ces pourcentages par le fait qu'en cas des lésions isolées, la réparation a été différée, alors que les lésions complexes ont été traitées en urgence. Concernant l’âge, les patients de moins de 22 ans ont obtenu 78,5% de bons résultats, contre seulement 33,3 % après 40 ans [20], la nature de la section qui été franche chez majorité de nos patient, constitue un facteur de bon pronostic. Dans le travail de V Berard [20] 71% de bons résultats ont été obtenu contre seulement 46 % lorsqu'il s′est agi d'un écrasement.

L′absence d′amélioration indiscutable de la qualité des résultats dans les séries récentes, malgré le soin apporté a la réparation des éléments anatomiques et à la rééducation, donne tout son intérêt aux recherches fondamentales sur la cicatrisation tendineuse [20].

## Conclusion

Au total, notre série présente des particularités que celle de la littérature notamment le mécanisme dominé par les agressions. Ses résultats sont moins bons que celle de littérature. Cependant le développement des techniques microchirurgicales avec mobilisation précoce ont amélioré les résultats fonctionnels.
